# Accuracy and clinical utility of near-point-of-care blood tests for predicting incident tuberculosis in exposed contacts in high burden settings: a multi-country observational cohort study

**DOI:** 10.21203/rs.3.rs-9126754/v1

**Published:** 2026-03-31

**Authors:** Shima M. Abdulgader, Arthur M. Chiwaya, Marc Banuls, Willy Ssengooba, Dinis Nguenha, James Sserubiri, Patricia Manjate, Catarina Bazima, Vinzeigh Leukes, Adam Penn-Nicholson, Achilles Katamba, Moses Joloba, Alberto L. Garcia-Basteiro, Grant Theron, Frank Cobelens

**Affiliations:** 1 DSI-NRF Centre of Excellence for Biomedical Tuberculosis Research; South African Medical Research Council Centre for Tuberculosis Research; Division of Molecular Biology and Human Genetics, Faculty of Medicine and Health Sciences, Stellenbosch University, Cape Town, South Africa.; 2 Fundación Privada Instituto de Salud Global Barcelona (ISGlobal), Spain; 3 Makerere University, Kampala, Uganda; 4 Centro de Investigação em Saúde de Manhiça (CISM), Maputo, Moçambique.; 5 FIND, Geneva, Switzerland.; 6 Centro de Investigación Biomédica en Red de Enfermedades Infecciosas CIBERINFEC, Barcelona, Spain.; 7 Amsterdam UMC, University of Amsterdam, Department of Global Health and Amsterdam Institute for Global Health Development, Netherlands

**Keywords:** Tuberculosis, Prediction, Contacts, Diagnostic Accuracy

## Abstract

**Background:**

The scale-up of tuberculosis preventative treatment is a global health priority yet constrained by our inability to identify which exposed contacts should be prioritised. We evaluated the diagnostic accuracy and clinical utility of new and established tests, including the first deployable host mRNA assay, done near point-of-care to diagnose incident tuberculosis in contacts in Mozambique, South Africa, and Uganda

**Methods:**

Contacts microbiologically-negative for tuberculosis or without symptoms were followed for 12 months. Xpert MTB-Host Response (Xpert MTB-HR), haemoglobin, and CRP was done by a minimally trained healthcare worker at M0 and M6 using finger-prick blood. Tuberculosis disease was identified at months 0, 6 and 12 by symptom- and chest X-ray-screening (microbiological testing if symptomatic or able to expectorate sputum or CXR-positive; CXR M12 only). Predictive accuracy and decision curve analyses were done, with microbiological confirmation serving as the primary reference standard.

**Results:**

Among 3,031 contacts, 66 incident cases occurred. At a 75% sensitivity threshold (the minimum WHO-recommended sensitivity), Xpert MTB-HR and haemoglobin had specificities of 55% (95% confidence interval 53–56) and 25% (24–26), respectively. CRP’s highest sensitivity with non-zero specificity was 52% (38–65) at 71% (69–72) specificity. At 75% specificity (the minimum WHO-recommended specificity), sensitivities were 61% (48–74), 37% (25–51) and 41% (28–55), respectively. Xpert MTB-HR and CRP in parallel (either positive) had highest sensitivity [76% (62–87)] and specificity of 59% (58–61), and the best clinical benefit for a number-willing-to-treat of 20–50.

**Conclusion:**

No tests individually met WHO sensitivity and specificity criteria but Xpert MTB-HR came closest. This work resets expectations for host RNA tests. Multi-marker strategies, including personalised risk scores, should be pursued.

## Introduction

One-quarter of the global population is infected with *Mycobacterium tuberculosis*. The risk of progression to disease is highest within the first two years after exposure; however, only 5–10% of infected individuals ever develop active tuberculosis (1, 2). Proactive strategies such as systematic screening of high-risk groups like contacts are essential to reduce progression to active disease and rationally allocate preventative treatment, thereby accelerating progress towards ending tuberculosis by 2035 (3).

Efforts to prioritize who receives preventive treatment are hindered by the absence of reliable tools to predict progression after exposure. While interferon gamma release assays (IGRA) and tuberculin skin test (TST) are widely used to determine immune sensitisation to *Mycobacterium tuberculosis*, they have limited positive predictive value (PPV) for progression (<5% over 2 years) (4, 5).

Recent advances in biomarker discovery, especially peripheral host transcriptomic signatures, hold promise for prediction. One of best performing signatures, Sweeney3, has a sensitivity and specificity of 57% (95%CI 45–69%) and 91% (89–93%) for incident TB within six months (6).This signature was subsequently adapted into the first-in-class Xpert MTB-Host Response (Xpert MTB-HR) test that uses the near-point-of-care and widely-deployed GeneXpert platform. However, Xpert MTB-HR evaluations to date have focussed on diagnosing tuberculosis, not predicting incident tuberculosis (7, 8).

C-reactive protein (CRP) is an established screening biomarker for tuberculosis in people living with HIV (PLHIV) and, in community-based surveys and outpatient studies enrolling adults regardless of symptoms, a threshold of ≥5 mg/L has 50–84% sensitivity and 61–72% specificity for tuberculosis (9, 10). However, it did not discriminate healthy household contacts from those with asymptomatic tuberculosis (11). To our knowledge, CRP has not been measured longitudinally in exposed contacts.

Similarly, haemoglobin may represent a useful predictor of incident tuberculosis as anaemia is a recognized clinical feature of tuberculosis. In PLHIV on antiretroviral therapy, anaemia is a strong short-term predictor of incident tuberculosis within four months (12). Evidence on its performance as a predictor of incident tuberculosis in people without HIV is however limited.

Despite promise, major knowledge gaps, such as a lack of comparative data, remain in translating biomarker discoveries into practical, scalable prediction tools for tuberculosis prediction. We therefore did a head-to-head multicountry evaluation of Xpert MTB-HR, haemoglobin and CRP for incident tuberculosis in exposed tuberculosis contacts in Mozambique, South Africa, and Uganda.

## Methods

### Ethics

The study (Clinicaltrials.gov NCT04825327) was done per the Declaration of Helsinki. In Mozambique, approvals were from the Centro de Investigação em Saúde da Manhiça Institutional Bioethics Committee for Health (CIBS-CISM/032/21) and the National Bioethics Review Board (390/CNBS/21). In South Africa, approvals were from the Stellenbosch University Faculty of Health Sciences Research Ethics Committee (M20/06/018), the City of Cape Town (6470), and the Western Cape Province (WC_202107_016). In Uganda, approvals were from the Makerere University School of Biomedical Sciences Research Ethics Committee (SBS-863) and the Uganda National Council for Science and Technology (HS1242ES).

### Eligibility

Index cases were eligible if they were ≥18 years of age, met the study definition of tuberculosis disease, provided written informed consent, and were willing to comply with study procedures, including supplying sputum samples for culture and identifying or referring eligible contacts. Contacts were eligible if they were ≥12 years old, met the study definition of a contact, and were able and willing to attend follow-up visits. They were required to provide consent or assent, supply sputum and blood samples, and agree to chest radiography at 12 months, with urine pregnancy testing for women of childbearing potential.

### Recruitment and follow-up

#### Index cases:

**Figure 1.**
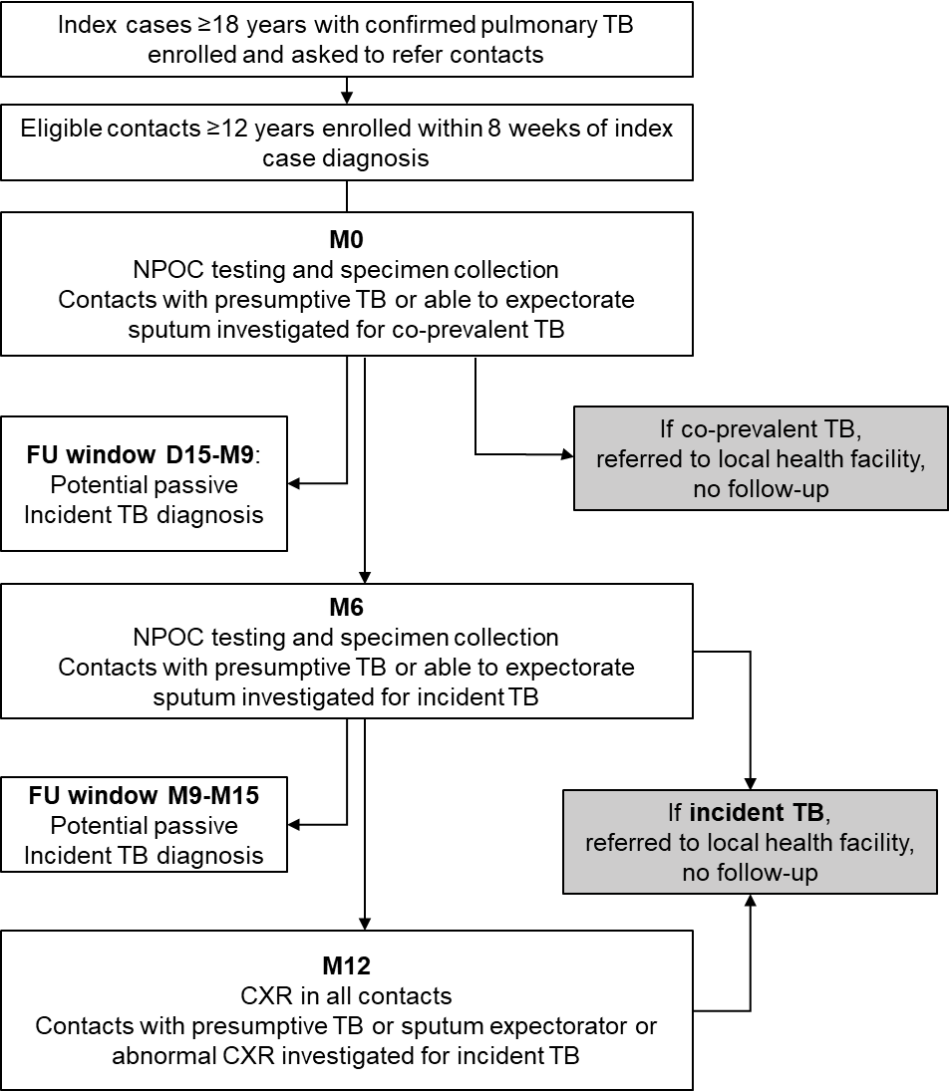
Study Design Enrolment, near point-of-care testing and follow-up of contacts. Recently diagnosed index cases were asked to refer contacts within eight weeks of their first positive TB result. Eligible contacts were screened for TB at recruitment and TB-negative contacts enrolled. Near point-of-care testing with the three candidate tests was done at M0 and repeated at M6 (light-shaded boxes). Contacts who met presumptive TB or sputum expectorator definitions were investigated for co-prevalent (at M0) or incident TB (at M6 and M12) and exited the study if positive (dark-shaded boxes). All contacts eligible for M12 had a CXR. **Abbreviations:** M0; month 0, M6; month 6, FU; follow-up, TB; tuberculosis, POC; point-of-care, CRP; C-reactive protein, Hb; haemoglobin, CXR; chest X-ray.

Index cases were enrolled within six weeks of their positive microbiological result. *Contacts:* Figure 1 illustrates contacts’ flow and study procedures. Contacts were enrolled within eight weeks of the date of specimen collection for the index case’s first positive result and followed for 12 months or until incident tuberculosis. Contacts attended three scheduled visits: baseline (M0), month 6 (M6), and month M12 with a three-month window on each side of M6 (range: M3-M9) and M12 (range: M9-M15) visits. Written informed consent was obtained from all participants (index cases or contacts), with parental or guardian consent for those 12–17 years. All study related data was captured and stored in REDCap (13).

### Study procedures

Near point-of-care testing was performed at M0 and M6. Contacts with tuberculosis-compatible symptoms or able to expectorate sputum spontaneously (**Supplementary Sections 1.1 and 1.2**) were investigated for tuberculosis at M0, M6, and M12 visits, but also were instructed to come to the study facilities in between if symptoms were present. At M12, all contacts underwent chest X-ray to rule-out tuberculosis disease (**Supplementary Section 1.7**).

### Specimen collection

#### Finger-prick blood:

At M0 and M6, 1–3 drops was collected and applied to each of the three tests. Additional drops were collected from contacts with unknown HIV status, or a negative HIV result older than three months and tested using a rapid HIV test.

#### Sputum:

Contacts with presumptive tuberculosis symptoms or meeting sputum expectorator criteria provided at least two sputa (≥1 mL each). If necessary, sputum induction was performed using nebulised 3 –10% NaCl.

### Near point-of-care testing

For each test, if the initial result was invalid (no value reported), the test was repeated once using a new specimen (same aliquot of serum used for CRP if done retrospectively No prespecified cutoff value for tuberculosis prediction was provided by any of the manufacturers.

#### Xpert MTB-HR:

100 μL blood was collected using a minivette with EDTA (SARSTEDT, Nümbrecht, Germany), added to the cartridge (Cepheid, Sunnyvale, USA) and loaded on the GeneXpert platform (14). We used the formula {cycle threshold (ct) [ct GBP5 + ct DUSP3]/2 – ct TBP} to generate the tuberculosis score (TBscore) (15).

#### CRP:

Testing using the LumiraDx platform (Roche Diagnostics, Basel, Switzerland) was performed using a combination of prospectively collected fingerprick blood and retrospective samples (stored serum) (16). The 20μL sample was then added to the test strip and loaded into the platform. Due to a disruption in test supplies, near point-of-care CRP testing was temporarily halted and later conducted retrospectively on frozen serum that we have separately shown to give similar results (17).

#### Haemoglobin:

100 μL blood was collected using microcuvette, placed into the Haemoglobin 801 Analyzer (HemoCue, Angelhom, Sweden) and tested per the manufacturer’s instructions (18).

### Sputum microbiology

WHO-endorsed protocols for sputum processing, smear microscopy, culture, and Xpert MTB/RIF Ultra (Xpert Ultra) were used. MGIT960 liquid culture (Becton, Dickinson and Company, Franklin Lakes, USA) was used across the study sites. In South Africa, GenoType MTBDR*plus* (v2.0; Bruker-Hain Life Sciences, Nehren, Germany) was done on culture-positive growth for *Mycobacterium tuberculosis* complex (MTBC) speciation and rifampicin resistance detection, while the Ag MPT64 rapid test used for speciation in Mozambique and Uganda. Xpert Ultra (Cepheid, Sunnyvale, USA) was done on the remaining sample (19).

### Data analysis

#### Primary outcomes and definitions

The primary outcome was the predictive accuracy of each test for MRS-positive incident tuberculosis, over six-months from M0 (days 15–274) or M6 (days 275–457) or both timepoints combined. Sensitivity analyses were done using extended (eRS) and stringent microbiological (sMRS) reference standards (**Supplementary Section 2**). All analyses used the MRS unless stated otherwise. We adhered to the Standards for Reporting of Diagnostic Accuracy Studies (STARD) (**Supplementary Table 13**) (20).

#### Secondary outcome

We evaluated predictive performance of baseline testing for incident tuberculosis over the full follow-up period (days 15–457).

#### Sensitivity analyses approach

##### Predictive performance based on predefined cutoffs

For all predictive analyses, we evaluated sensitivity and specificity using predefined cut-off values for each test with binomial 95% confidence intervals (CIs). For Xpert MTB-HR, we applied a TBscore threshold of ≤–1.275 (Xpert MTB-HR_≤–1.275_), as described for co-prevalent tuberculosis in symptomatic adults (15). For CRP, we used 10 mg/L (CRP_≥10_) and 5 mg/L (CRP_≥5_) recommended by the WHO to screen for co-prevalent TB. All CRP results reported as <5 mg/L were assigned an arbitrary value of 2.5 mg/L. For haemoglobin, we used WHO-defined anaemia definitions (**Supplementary Section 1.8**).

##### Predictive performance based on Receiver Operator Characteristic Curves

For each test and outcome, we performed Area Under the Receiver Operator Characteristics (AUROC) analyses, selecting thresholds that corresponded to the minimum recommended WHO sensitivity (75%) and specificity (75%) for a tuberculosis prediction test (21). As an exploratory analysis, we also assessed whether changes in test quantitative readouts between M0 and M6 had predictive value (**Supplement Section 3.1**).

#### Predictive algorithms

We in addition evaluated two-test predictive testing algorithms: (1) parallel positive, where a positive result on either test was considered predictive, and (2) sequential positive, where both tests needed to be positive. We tested combinations of the three tests at the same prespecified cutoffs used for single tests. For this multiple test analysis, we report only the best-performing predictive algorithms based on sensitivity and specificity.

#### Decision curve analysis

We performed decision curve analysis (DCA) to evaluate the clinical utility of testing strategies across a range of threshold probabilities (22).

All analyses were done using Stata Statistical Software (v18; StataCorp LLC, USA). AUROCs were compared for subgroups using the *roccomp* command in Stata. Visuals were generated in Stata, R (v4.5.1; R Foundation for Statistical Computing, Vienna, Austria) and GraphPad Prism (v8; GraphPad Software, USA).

## Results

### Recruitment and retention

**Figure 2.**
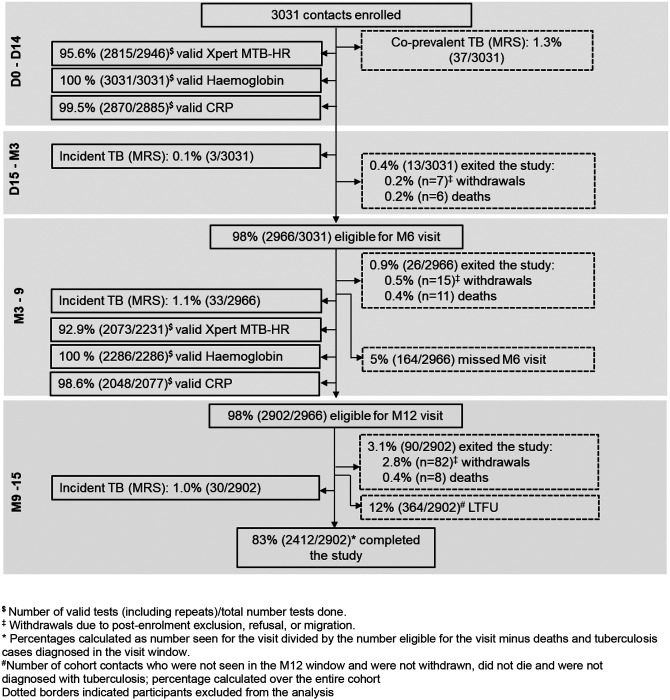
Participant flow and retention

Between 21 April 2021 and 31 January 2025, we enrolled 1291 index cases and 3031 contacts of whom 1107 (36%) were enrolled in Mozambique, 1055 (35%) in South Africa, and 869 (29%) in Uganda (**Supplementary Table 1**). A total of 110 withdrawals and 25 tuberculosis-unrelated deaths occurred. By the end of M12 follow-up window 2402/3031 (79%) contacts completed follow-up without progressing to incident tuberculosis, and 364/2902 (12%) were lost to follow-up (i.e., missed the M12 visit) (Figure 2).

### Baseline characteristics of contacts

The median age [interquartile range (IQR)] was 29 (19–44) years. Of the 3031 contacts, 19% were adolescents, 60% (1841/3031) female, 15% (449/3031) were PLHIV, and 9% (276/3031) had at least one prior tuberculosis episode (**Supplementary Table 2**). 43/3031 (1.7%) had co-prevalent tuberculosis.

### Characteristics of contacts with incident tuberculosis

We detected 93 cases with a median (IQR) day from enrolment to collection of specimens for diagnosis of 266 (IQR 173–362; **Supplementary Figure 1**). Of these, 66 (71%) were MRS positive: 12 (18%) Ultra- and culture-positive, 54 (82%) were Ultra-positive only. Forty-two (64%) incident cases were diagnosed during study visits, while 24 (36%) were identified through routine care. Twenty-five (29% of 93) incident cases were clinically diagnosed including 12 at M12 and 13 between study visits through routine care (**Supplementary Figure 2**). Compared to those without incident tuberculosis, contacts with incident tuberculosis were older with 38 (IQR 20–50) vs. 28 (19–44) years, more likely living with HIV [21% (14/68) vs.15% (426/2920)] and more often previously tuberculosis-treated, however, they were, at baseline, not sicker than those without incident TB (Table 1, **Supplementary Table 2**).

### Six-months predictive performance

**Figure 3.**
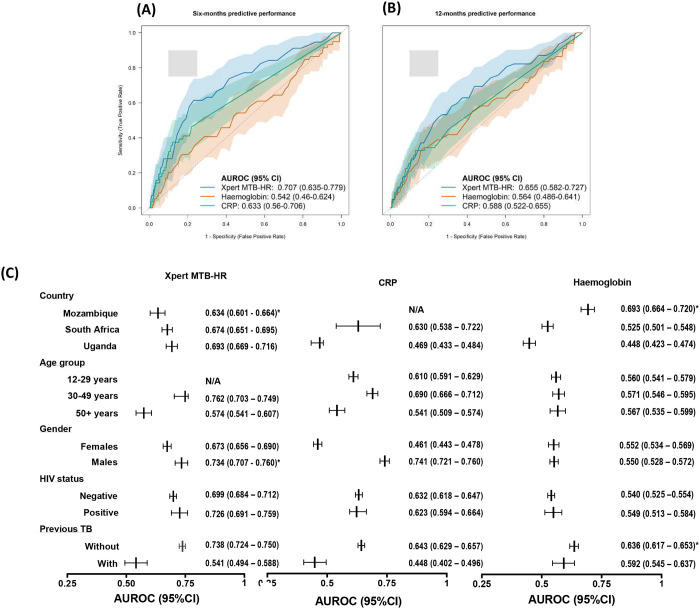
Predictive performance of Xpert MTB-HR, C-reactive protein, and haemoglobin. Areas under the Receiver-Operator Characteristics curves (AUROC) of the three near point-of-care tests for prediction of incident tuberculosis amongst contacts according to the microbiological reference standard. **(A)** Prediction over 6 months from month 0 or month 6. **(B)** Prediction over 12-months. The shaded area for each line represents the 95% confidence interval. The grey shaded square corresponds to WHO minimum and optimum sensitivity and specificity TPP thresholds **(C)** AUROC of the three tests stratified by key clinical subgroups.

**Figure 4.**
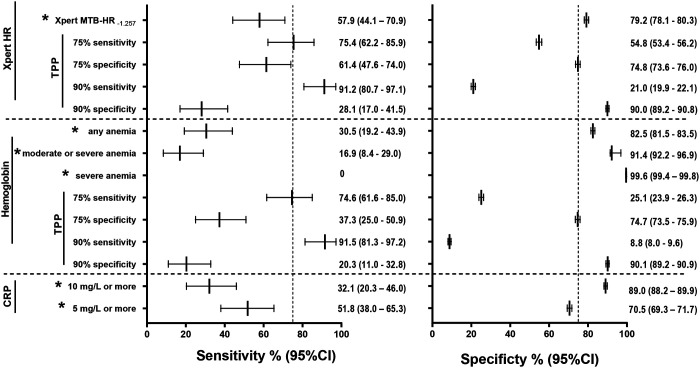
Six-months predictive performance at prespecified cut-offs and Target Product Profile-based performance thresholds. Sensitivity and specificity estimate with 95% confidence intervals for each test for incident tuberculosis within six months from M0 or M6. For all three tests prespecified (*) cutoffs (Xpert MTB-HR_≤-1.257_, C-reactive protein (CRP_≥10_) or CRP_≥5_, WHO anemia definitions, and the WHO Prediction Test Target Product Profile (TPP) thresholds were used. The vertical dotted line indicates the minimum (75%) TPP sensitivity and specificity cutoffs.

At a prespecified threshold of Xpert MTB-HR–_1.275_, sensitivity was 58% (44–71%) and specificity was 79% (78–80%), yielding an AUROC of 0.707 (0.635–0.779). Anemia showed a sensitivity of 30% (19–44%) and specificity of 82% (81–84%), whereas moderate or severe anemia improved specificity to 92.2% (91–97) at the expense of sensitivity (17%; 8–29%); the corresponding AUROC was 0.542 (0.460–0.624). CRP demonstrated high specificity at conventional cutoffs; 89% (88–90%) at ≥10 mg/L and 70% (69–72%) at ≥5 mg/L, albeit with lower sensitivity; the AUROC was 0.633 (0.560–0.706) (Figure 3A, Figure 4).

For all tests, AUROCs for six-months prediction were similar when only M0 data (i.e., without repeat testing at M6), were used (**Supplementary Tables 4, 6, and 11**).

#### Performance against the WHO target product profile

At the test threshold corresponding to the minimum TPP specificity requirement of 75%, Xpert MTBHR at ≤–1.275 achieved a sensitivity of 61% (48–74). While, at the same 75% specificity threshold, haemoglobin demonstrated a sensitivity of 37% (25–51), and at the corresponding 75% sensitivity threshold, its specificity was only 25% (24–26). None of the AUROC-derived sensitivity–specificity combinations for all three tests met the TPP minimum performance requirements for a predictive test (Figure 4).

#### Performance against secondary reference standards

Accuracy estimates based on alternative reference standards are in **Supplementary Figure 3** and **Supplementary Table 11**. Overall performance was like that observed using the MRS

#### Performance by clinical stratifications

The AUROCs for Xpert MTB-HR did not differ by site or HIV status; however, they were higher among contacts aged 30–49 years and those with no history of tuberculosis. In contrast, sensitivity and specificity for anemia or CRP did not vary across subgroups (Figure 3C, **Supplementary Tables 5, 7 and 10**).

### Predictive performance of biomarker trajectories over six months

The AUROC for the change in Xpert MTB-HR (ΔHR) was 0.626 (0.516–0.735), which was lower than the AUROC for the M6 measurement alone (0.755; 0.736–0.773, p=0.048) (**Supplementary Table 4**). There were no significant differences for CRP, where the AUROC for ΔCRP was 0.587 (0.443–0.733) compared to 0.678 (0.564–0.792) for M6 (p=0.126), or for haemoglobin where the corresponding AUROCs were 0.578 (0.374–0.634) and 0.504 (0.444–0.711, p=0.262). At a threshold of the magnitude of change in each test that corresponded to the WHO-recommended sensitivity, no specificities met WHO criteria and vice versa (**Supplementary Table 12, Supplementary Figure 4**).

### Twelve-months predictive performance for baseline near point-of-care testing

M0 results yielded AUROCs of 0.655 (0.582–0.727) for Xpert MTB-HR, 0.564 (0.486–0.641) for haemoglobin, and 0.588 (0.522–0.655) for CRP all lower than their six-month predictive performance (Figure 3). Sensitivity and specificity at prespecified cut-offs and TPP thresholds were like or lower than those observed for six-month prediction (**Supplementary Figure 5**)

### Maximising performance through predictive algorithms

For parallel near point-of-care testing, a positive result from either Xpert MTB-HR–_1.275_ or CRP_10_ provided the highest sensitivity of 76% (62–87%), meeting the minimum WHO TPP sensitivity criteria at the expense of specificity (59%; 58–61%) (**Supplementary Table 13**). Combining moderate-to-severe anemia with CRP_5_ yielded 44% (31–59%) sensitivity, although specificity remained ≥0.80. For all sequential combinations, specificities were very high (range: 88–98%), while sensitivities ranged from 6% (1–15%) to 38% (26–53%).

### Clinical utility assessment using Decision Curve Analysis

**Figure 5.**
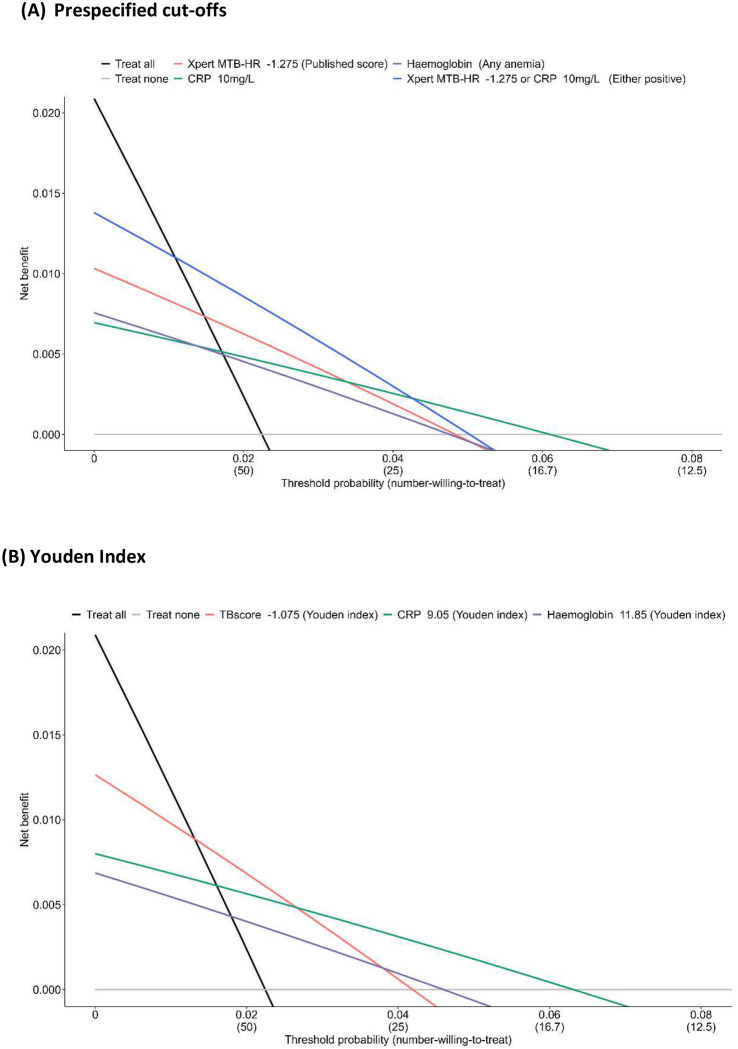
Decision curve analysis of Xpert MTB-HR, haemoglobin, and CRP. Decision curve analysis in which each test is compared with default strategies of treating all or no people **(A)** using prespecified cutoffs along with the best performing two-test algorithm and **(B)** the optimized cutoffs based on the Youden index for each test. Threshold probability is the risk of tuberculosis disease at which a clinician or patient would opt for preventive treatment and is the reciprocal of the number-needed-to-treat to prevent a single incident case. Net benefit is calculated at a range of threshold probabilities as the true positive rate minus a weighted false positive rate, in which the weighting is the threshold probability.

Decision curve analysis (Figure 5) demonstrated that Xpert MTB-HR–_1.275_ provided the greatest net benefit among single-test strategies at lower thresholds (~2–3.5%), whereas CRP_10_ performed best at higher thresholds (~3.5–6%), reflecting the trade-off between sensitivity and specificity as threshold probability increases and tolerance for overtreatment decreases. Net benefit for haemoglobin was consistently lower than for the other biomarkers evaluated.

For threshold probabilities between 2% and 5%, corresponding to a number-willing-to-treat with preventative treatment of 20 to 50 exposed contacts, several biomarker-guided strategies provided greater clinical value than treat-all or treat-none approaches. Across this range, parallel Xpert MTB-HR–_1.275_ or CRP_10_, in which individuals positive on either test were offered preventative treatment, consistently yielded the highest net benefit and outperformed either marker alone up to a threshold probability of approximately 4.2% (number-willing-to-treat ~24) and was favourable to treating none up to a threshold of 5%. A similar pattern was noted at the Youden index-derived thresholds for all three tests.

## Discussion

In this multi-country evaluation of near point-of-care testing for TB prediction, including the first-in-class host transcriptomic test, (1) for six-month prediction when sensitivity or specificity was fixed at WHO-recommended values, the corresponding sensitivity or specificity did not reach the WHO-recommended value for each of the three tests. Xpert MTB-HR was closest, (2) combined testing using Xpert MTB-HR with CRP in parallel met the minimum WHO TPP sensitivity criterion at the expense of specificity; and (3) if a number-willing-to-treat between 20 and 50 people is considered acceptable, using the combined Xpert MTB-HR and CRP algorithm to guide preventative treatment would have the best clinical benefit compared to either treating all or none. Together, these data have implications the prioritisation of preventative treatment in people exposed to TB.

Xpert MTB-HR performed similarly to pooled analyses by Gupta et al. (6), which reported AUROC ~0.72 for six-month prediction when host RNA was measured using laboratory-based methods. Our study demonstrates this signature can be deployed in a near point-of-care format, though cost considerations remain critical for implementation. The fact that, no matter the threshold used, sensitivity and specificity did not meet WHO criteria, suggests the signature measured by Xpert MTB-HR is alone an inadequate predictor of incident TB and that, as it was the promising of the previously reported signatures, host mRNA cannot meet WHO performance benchmarks.

At CRP_5_ and CRP_10_, CRP achieved specificity ≥70% and a highest sensitivity of 52%. In comparison, IGRA and TST typically achieve specificities of ~74–76% for predicting progression to tuberculosis disease, but their sensitivities remain modest (63–65%) (23, 24). While CRP’s predictive accuracy is limited, its operational simplicity requiring only a finger-prick sample and delivering results within minutes - a distinct advantage over IGRA and TST.

Anemia showed high specificity but low sensitivity for predicting incident tuberculosis, limiting its utility as a standalone marker, in contrast to prior predictive studies in PLHIV (12, 25). These differences likely reflect variation in population characteristics and study design, where dynamic monitoring and immunosuppression in PLHIV cohorts may amplify predictive value (12, 25). Thus, while haemoglobin may serve as an adjunct marker in high-risk groups, its role in general household contacts appears limited.

Performance remained consistent across definitions of incident tuberculosis, indicating robustness to variations in reference standards. Importantly, performance did not differ across key subgroups including study site, HIV status, gender, and other demographics, supporting potential applicability in diverse settings.

Despite operational advantages, none of the near point-of-care tests met, at single threshold, both the WHO TPP minimum sensitivity and specificity of 75%, limiting their utility as standalone tools for initiating risk-based TPT. Across clinically plausible treatment thresholds (2–5%), a parallel Xpert MTB-HR and CRP testing strategy offered the greatest clinical benefit for targeting preventive tuberculosis treatment, outperforming either test alone and both treat-all and treat-none approaches. However, no approach achieved a PPV >6%; hence, ‘treat none’ was the best strategy above this threshold.

Our study had limitations. First, tuberculosis evaluation relied on symptom screening or ability to expectorate sputum. This reflects real-world practice in the settings where predictive tests are likely to be implemented. This limitation is partly mitigated by the fact that many asymptomatic contacts with tuberculosis may later revert to a healthy state, the clinical significance of this remains uncertain, along with our multiple follow-ups and the inclusion of chest X-ray at the end of the study. Second, we included Ultra-trace-positive results to increase incident case detection and because untreated trace-positive contacts have been linked to higher risk of tuberculosis progression (10). However, applying a more stringent microbiological reference standard did not affect our findings. The use of tests with a higher LOD for CRP may have uncovered thresholds below 5 mg/L that may be useful for screening, however, these are not NPOC. Finally, only a limited proportion of MRS-positive incident cases were culture-positive. This partially reflected diagnosis outside study visits where treatment had already been started before a specimen for culture could be taken. In addition, discordant Xpert-culture results are common in active case finding and related to low bacterial burden (11).

In conclusion, near point-of-care Xpert MTB-HR, haemoglobin-defined anemia, and CRP showed modest predictive performance for TB progression and did not meet WHO TPP criteria for a prediction test. Xpert MTB-HR accuracy remains insufficient for standalone use in risk-based TPT. Although Haemoglobin and CRP have operational advantages, they had limited utility individually but may serve as adjuncts in high-risk populations, and combining CRP with Xpert HR testing may enhance clinical utility compared to single-marker approaches.

## Supplementary Material

Supplementary Files

This is a list of supplementary files associated with this preprint. Click to download.

- SupplementPreFIT12March2026.docx

## Figures and Tables

**Table 1.** Baseline clinical characteristics of contacts diagnosed with tuberculosis. Data are median (IQR) or n (%).

Characteristic	Overall^#^ (n=2988)	Contacts diagnosed with bacteriologically confirmed incident tuberculosis (n=68) **	Contacts without incident TB (n= 2920)
Age in years	29 (19–44)	38 (30–50)	28 (19–44)
Adolescents (12–17 yrs)	583 (19)	2 (3)	581(20)
Females	1820 (60.7%)	35 (52)	1785 (61)
Symptoms*	-		-
Cough	-	38 (56)	-
Chest pain	-	19 (28)	-
Night sweats	-	30 (44)	-
Weight loss	-	22 (32)	-
Fever	-	13 (19)	-
Met symptoms definition	341 (11)	21 (31)	320 (11)
Able to expectorate sputum	457 (15)	45 (66)	412 (14)
Morbidity score (median (IQR))	3 (2–3)	3 (2–3)	3 (2–3)
HIV positive	440 (15)	14 (21)	426 (15)
At least 1 previous tuberculosis episode at baseline	258 (9)	20 (29)	238 (8)
Type of contact			
Household contact	2800 (94)	62 (91)	2738 (94)

# Excluding co-prevalent tuberculosis cases diagnosed within 14 days post-enrolment.

* At the time of sample collection that used to diagnose tuberculosis. Symptom information was missing for 23 contacts overall, including 16 bacteriologically confirmed cases.

** Includes one case Xpert Ultra Trace positive only with a history of tuberculosis treatment within the preceding 2 years and one case with positive urinary LAM only.

Morbidity score was calculated using cough, dyspnea, chest pain, anemia, body mass index and middle-upper arm circumference. Each contact is scored out of 7(27). IQR: Interquartile range.

## Box: Definitions (see more details in Supplementary section 1)

**Index case:** *Bacteriologically confirmed pulmonary tuberculosis patients diagnosed in routine care*.

**Contacts:** *Any person ≥12 years exposed to an index case enrolled in the study*.

**Tuberculosis disease:** *Any pulmonary tuberculosis diagnosed in study participants based on a microbiological reference standard*.

**Microbiological Reference Standard:** *A positive sputum culture or a positive sputum Xpert MTB/RIF or Xpert MTB/RIF Ultra result*.

**Co-prevalent tuberculosis:** *Tuberculosis diagnosed in a contact from a specimen taken within 14 days post-enrolment*.

**Incident tuberculosis:** *Tuberculosis disease detected after day 14 in a contact who did not have co-prevalent tuberculosis*.

**Passive tuberculosis detection:** *Tuberculosis disease diagnosis that occurred outside scheduled study visits with testing done by study staff or programmatic health services*.
